# The influence of ultraviolet radiation on aflatoxin producing *Aspergillus* species' isolated from Iranian rice

**DOI:** 10.1016/j.toxrep.2022.07.007

**Published:** 2022-07-12

**Authors:** Hamed Faraji, Farideh Tabatabaee Yazdi, Nematollah Razmi

**Affiliations:** a Department of Microbiology, College of Sciences, Agriculture and Modern Technology, Shiraz Branch, Islamic Azad University, Shiraz, Iran; b Department of Food Science Industry Faculty of Agriculture, Ferdowsi University of Mashhad, Iran

**Keywords:** Aflatoxins, UV-irradiation, HPLC, Aspergillus

## Abstract

Cereal grains are a favorable habitat for aflatoxin- producing fungus to develop. the current investigation was carried out to evaluate the quantity and kind of contaminated imported grains and rice generated in the province of Shiraz, Iran. A total of 60 random rice samples were taken from paddy fields in October and November 2020. *Aspergillus* genera were detected using PCR. HPLC was used to determine the quantity and type of aflatoxin and mycotoxins in samples collected. Irradiation studies were carried out utilizing a collimated beam system with wavelengths ranging from 200 to 360 nm. The quality of rice was assessed using UV light therapy on some of the changed factors, such as amylose content, aroma, and brightness [*P* < *0.05*]. *Aspergillus* genera were found in 33.3% [20 samples of 60] of rice samples after morphological and molecular analysis of the ITS gene. According to the sequencing experiment, 12 strains [60%] were identified as *Aspergillus flavus*, whereas 8 strains [40%] were identified as *Aspergillus parasiticus*. *Ver-1* and *afl-R* genes were positive in 12/12 [100%] *Aspergillus flavus* and 87.5% in *Aspergillus parasiticus.* According to the HPLC findings, three *Aspergillus parasiticus* strains [37.5%] were able to create all four types of aflatoxins, and aflatoxins B1, B2, G1, G2 were produced by 16.6% of *Aspergillus flavus* strains. Aflatoxin-1 (AFG1) was lowered to 35.1, 48.2, 59.9, and 65.2%, significantly, at doses of 1.22, 2.44, 3.66, and 4.88 Jcm^−2^ [*P* < *0.01*]. Furthermore, at doses of 1.22, 2.44, 3.66, and 4.88 Jcm^−2^, AFB2 and AFG2 was shown to be reduced by 13.1%, 11.7%, 30.3%, and 28.9%. [*P* < *0.05*]. At a maximum dose of 4.88 Jcm^−2^, AFB1 was shown to be extremely susceptible to UV irradiation, with a > 70% decrease seen [*P* < *0.001*]. Our findings imply that UV irradiation with lower energy and lower danger can help minimize aflatoxin contamination in food.

## Highlights

• Aspergillus Isolates from Rice were Identified.
• UV light decreased Aflatoxin from rice.
• The qualitative effects of UV radiation on rice have not revealed any substantial alterations or flaws in rice quality.

## Graphical Abstract

**Figure:**
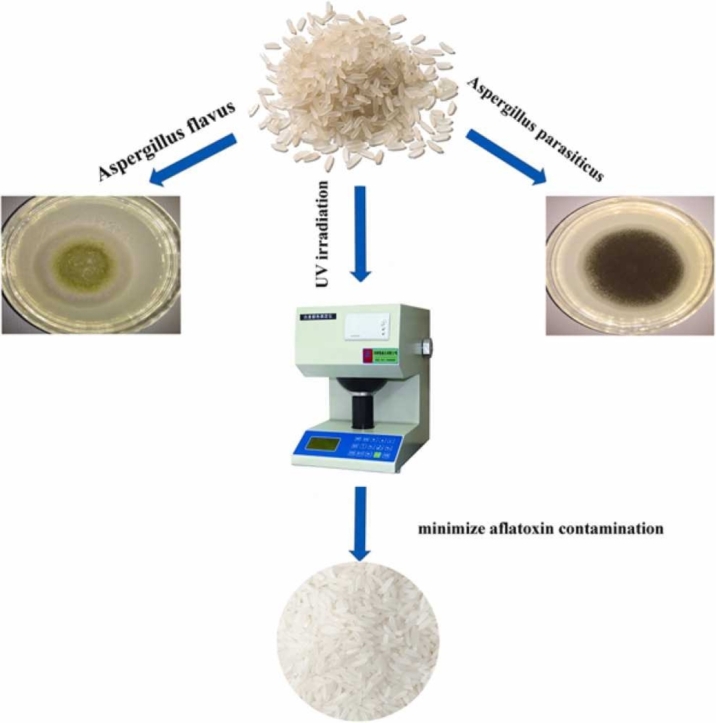

## 1 Introduction

Mycotoxins are fungus compounds that are regularly found in foods and can be harmful to the customer's health. Mycotoxins have serious maximum amounts approved in food across the globe; nonetheless, the longevity of mycotoxins or their compounds is a large safety problem, particularly in poor nations [1]. According to the Food and Agricultural Organization, almost a quarter of all grain harvests in poor nations are infected by mycotoxins. Aflatoxins are found in tropical and subtropical climates, in which the humidity levels circumstances are ideal for fungal development and the generation of these toxins. Such infection is common in agricultural products, particularly grain and rice-based meals [2]. Because these compounds are very stable and biomagnified in the food web, uptake of aflatoxins or their compounds are commonly recorded in crops, and their existence has been documented in meat, milk, water, and soil. Aflatoxins can also infect foods including grains, fruits, walnuts, spices, and by-products [3].

Aflatoxins are a class of powerful toxins that are cytotoxic, carcinogenic, mutagenic, hepatotoxic, and autoimmune problem. They are important because of their widespread distribution and adverse impacts on animals and human health [4]. *Aspergillus genera* generate aflatoxins, which are natural compounds. These microorganisms may be found in a variety of nations, particularly in tropical and subtropical areas, where the warmth and dampness are ideal for fungal development and toxin synthesis [5]. Both chronic and acute hepatocellular damages have been linked to aflatoxins consumption. Although AFB1, AFB2, and AFG1 are detected as pollutants in land-based foods, their compounds [AFM1 and AFM2] are present in grain-based foods such as diary, rice and wheat [6]. Rice is the most important food in Asian countries, especially in Iran. Rice as food is usually raped by fungi called *Aspergillus flavous* and *Aspergillus paraziticus* [7].

As a result, based on the kinds of meals ingested, individuals are at significant risk of falling victim to various forms of aflatoxins. Because of the link between AFB1 consumption and hepatocellular carcinoma, the Intergovernmental Agency for Research on Cancer [IARC] has placed AFB1 in the most hazardous category of carcinogens [group 1], according to the International Agency for Research on Cancer [IARC] [8].

Mycotoxins' strong thermal durability renders these compounds resistant to extreme temperature, raising the danger of exposure levels. In reality, various approaches for lowering aflatoxins concentrations have been identified [9]. These include greater heating, ultraviolet, light grinding, scrubbing, and the use of bio-sorbents or compounds, acid-base impact, oxidant impacts, or different inorganic and organic compounds [10]. The majority of research has looked into using a pulsed light [PL] generator to minimize aflatoxins [11]. However, current research suggests that ultraviolet [UV] light may be a more effective method for lowering aflatoxin, which has to be researched further.

UV irradiation is a decontamination method that eliminates a wide range of bacteria and may also be used to remediate chemical contaminants through straight photolysis and advanced oxidation [12]. Due to mycotoxins' photosensitivity, UV irradiation has long been recognized to be an efficient physical means for their elimination. A low-pressure light was utilized in this investigation by the researchers. Aflatoxins have a UV absorbance peak of 320 nm, while low-pressure mercurial lights have a maximal emission spectrum of 253.7 nm. This discrepancy between the poor lamp's peak absorption spectrum and aflatoxins' absorbing peaks might illustrate why aflatoxins are only a little degraded by UV-C exposure [13].

Some researches show UV irradiation is a low-risk or safe method for the degradation of aflatoxins [14], [15]. The optimized condition of UV irradiation is the potential that could be used on a large scale for industrial food processing. According to the studies, UV irradiation could have a risk for food and nutrient [16], [17].

One of the most important problems is the production of peroxide of fatty acid that is not considered in rice because rice has a very low level of fatty acid and lipids. Physicochemical properties like amylose percent and humidity of rice change after irradiation but it is not remarkable [18], [19]. Often reported UV consequence on lipids and this effect on rice isn’t important as low lipid content. The other aspect of irradiation risk is free radical formation; changes in biochemical characteristics of rice and production of high energy compounds like Hydrogen peroxide (H2o2) and accelerate oxidational reactions [20]. In this study, we show how UV irradiation may improve rice quality and flavor while reducing microbial contamination and aflatoxin exposure. For qualitative evaluation, the photodegradation of aflatoxin in the rice matrix was assessed, as well as other physical-chemical parameters such as brightness, flavor, and amylose were done.

## 2 Material and methods

### 2.1 Rice samples collection

A random sampling technique was used to obtain 60 samples of rice from Shiraz in November 2020. Approximately 300 g of rice were collected and transported in sterile packets to the laboratory.

### 2.2 Aspergillus colonies isolation of rice samples

Ten grams of rice seeds were randomly chosen from each collection. The chosen samples were washed and then cultured on Sabouraud dextrose agar [SDA]. According to morphological appearances and reproductive system, *Aspergillus* samples were isolated. Aspergillus fungi were recognized using microbiology and physiological procedures. Briefly, the samples were incubated into a growth media remaining 30 g/L sucrose, 3.0 g/L sodium nitrate, and di-potassium hydrogen phosphate (pH 6.0). The samples were incubated for 5 days at 30 ± 2 °C. fungal pathogens were collected, and the isolated fungi were injected in Erlenmeyer flasks separately and shaken on a shaker incubator at 150 rpm for 5 days at 30 ± 2 °C. The sample was kept at 2–8 °C for further use after culture [18]. The conventional method of classifying *Aspergillus* genera based on morphological recognition is challenging and can contribute to misdiagnosis, particularly for *Aspergillus niger* taxa, that are physically identical [21], [22].

### 2.3 ITS Sequencing for Aspergillus isolate confirmation

A) DNA Extraction and Mycelium Synthesis in Fungi
  The mycelium bulk was recovered in SDB media, rinsed with deionized water, and dried. Liquid nitrogen was used to powder the mycelium bulk. DNA extraction was performed according to cinna-colone extraction kit [Iran]. The process was carried out according to the manufacturer's instructions. With the use of phenol, chloroform, and isoamyl alcohol, mycelium powder was separated and polypeptide destroyed in three steps. Finally, ethanol solution was used to obtain the DNA, which was then rinsed in 70% ethanol [28]. The extracted DNA's quality [A260/A280] and quantity were then tested [NanoDrop, Thermo Scientific, Waltham, MA, USA].
B) Amplification of the ITS Gene

To amplify roughly 500 bp of *ITS* sequences, the ITS1-F and ITS4-R primer pairs [Table 1] were utilized. In a thermal-cycler [Biorad], the process was carried out using the following thermal program: 3 min at 95 °C, followed by 35 cycles of 95 °C for 45 s, 52 °C for 45 s, 72 °C for 1 min, and a 5-minute extension step at 72 °C. On a 2% agarose gel dyed with ethidium bromide [0.5 g/ml], the DNA's veracity was evaluated [Thermo Fisher Scientific, St. Leon-Rot, Germany]. The standard strain of *Aspergillus flavus* NRRL32354 was used to confirm and control the process [28]. Sequencing of PCR results was performed by GENEWIZ company [Germany].

**Table 1:** Details of oligonucleotide primers used for PCR and real-time PCR.

**Target**	**Primers** **Name**	**Sequences [5′= >3′]**	**Annealing** **Temperature [°C]**	**Product** **length [bp]**
*ITS*	ITS1-F ITS4-R	TCCGTAGGTGAACCTTGCGG TCCTCCGCTTATGATATGC	58	500
*ver1*-PCR	*Ver-1*-F *Ver-1*-R	GCCGCAGGCCGCGGAGAAAGTGGT GGGGATATACTCCCGCGACACAGCC	58	600
*afl-R*-PCR	*afl-R* -F *afl-R* -R	TATCTCCCCCCGGGCATCTCCCGG CCGTCAGACAGCCACTGGACACGG	62	120

### 2.4 Analyzing and identifying mycotoxins

A) Molecular analysis of aflatoxin-producing genes
  A PCR thermal cycler [Eppendorf Co., Hamburg, Germany] was used to execute the polymerase chain reaction [PCR] according to the Piri-Gharaghie et. al protocol [23]. Utilizing particular primers, the *ver-1* and *afl-R* genes, whose products are critical in aflatoxin production, were generated. Table 1 lists the primers that were utilized. The PCR values were tuned based on the size, melting temperature, and predicted ultimate product size, using a reaction solution volume of 25 μl.
B) The Quantity and Variety of Mycotoxins

Purification and identification were carried out using an immunoaffinity column [manufactured by Neocolumn in France under the brand name Neogene, which was obtained from its Iranian distributor "Sina Medicine Chemistry"] in conformance with National Standard 6872 and AOAC No. 9990, using an HPLC device [HPLC system Made by American Agilent Company, equipped with autosampler and fluorescence detector]. Toxin purification utilizing immunoaffinity columns with monoclonal antibodies against aflatoxins B1, B2, G1, G2 was done and high-performance liquid chromatography was used to assess aflatoxin levels in the *Aspergillus* species [13], [28]. HPLC results were evaluated following Iranian National Standard No. 6872. [ISIRI-6872].

Solvent extraction was used to get the toxin from the samples [methanol 80%]. After filtering through a sinter filter, the extract was diluted using Watman filter paper to a specific concentration. The antigens present in a sample were attached to particular antibodies in the immunoaffinity row [Zearala Test TM, Afla Test TM, Ochra Test TM, DON Test TM] at a bead per second rate. By comparing the standard substrate surface with an unknown specimen and taking into consideration the dilution factor in ng/g, injection, separation, recognition, and identification of mycotoxins were computed using reversed-phase HPLC columns and derivative and fluorescence detectors [HPLC device, Agilent, USA]. Infused samples were scanned and recovered in the same way that normal samples were in order to measure the recovery rate of aflatoxin toxins at a dosage of 5 ng.g^−1^. Aflatoxins were recovered at rates ranging from 70% to 110%. This value is appropriate by national requirements, indicating that the extraction processes were completed successfully.

### 2.5 Standards for aflatoxin

Aflatoxin levels in distilled water [Milli-Q] were developed to assess the breakdown of aflatoxins. The amounts of AFB1, AFB2, AFG1, and AFG2 in standard solutions were 1, 0.60, 0.62 and 1 μg/ml, respectively. Before Ultraviolet irradiation, the solutions were dissolved in distilled water. The ultimate solution quantity was kept below 5% in all situations. In UV irradiation studies, the final concentrations of AFB1, AFB2, AFG1, and AFG2 were 0.69, 0.46, 0.50, and 0.52 μg/ml, respectively [28].

### 2.6 Laboratory-collinear beam device

UV-therapy was carried out in quiescently stirred 10-ml beakers utilizing bench-scale quasi- collimated beam systems. A 1000-W UV light transmitting via 1 in. circle lens was installed to the MPUV-collimated beam system [Calgon Carbon Corporation Inc., Pittsburgh, PA, USA]. An IL-1700 radiometer with a validated SED 240 sensor and a W-diffuser was used to evaluate irradiation at the fluid surface [International Light, Peabody, MA]. The radiometer was used to estimate incoming irradiation during all investigations. According to Beer's law as per Bolton and Linden, UV treatment [or fluence] was determined by considering a range of parameters [reflection, sample depth, UV transmittance, and Petri factor] [24]. The emission wavelengths around 200 and 360 nm were used to calculate the UV radiation supplied for MPUV. The median UV irradiation throughout the wavelength range was used to estimate the UV radiation. The median antimicrobial strength in a solution is the volume-averaged strength in the antimicrobial range, adjusted for the liquid's uptake.

Each procedure was given in triplicates, with 5 ml of sample solution placed into a 10-ml beaker for UV irradiation. The treatment period for each UV dosage was estimated according to Bolton and Linden's guidelines [24]. In a continuously stirred batch process, specimen absorbance was read using a Cary 100 Spectrophotometer linked to a single infinite plate [Agilent Technology, Santa Clara, CA, USA] and the wavelength was used to estimate mean irradiation. To achieve particularly exposed periods, the median irradiation was reduced by the desired UV dosage. Petri factor was used to adjust for non - homogenous light output [24]. Maximum UV-C dose [Eqs. 1) and (2)] was supplied to study liquids at 0, 1.22, 2.44, 3.66, and 4.88 J cm^−2^, correspondingly, for time values of 10, 20, 30, and 40 min. During larger doses of irradiation, the temperature was adjusted with a water bath.

(1) Average Fluence rate [mW·cm^−2^] = Incident Fluence × [1–10^-[a×d]^ / ln10 × a × d] × [ L/ L+D]

(2) UV dose [mJ⋅ cm^−2^] = Average fluence rate × Treatment time [s]

Incident fluence is the incoming irradiation at the liquid's interface, [a] is the absorption coefficient per each spectrum, [d] is the fluid's thickness in the beaker, and [L] is the length between the light's core and the lower meniscus of the fuel's surface. This equation was tweaked to fit the trial circumstances. Because the area of the light source is bigger than the surface area of the sample in the beaker, the Petri factor has been removed from the equation above [13].

### 2.7 Evaluation of mycotoxin degradation utilizing HPLC

For the study of AFB1, AFB2, AFG1, and AFG2 aflatoxins, an Agilent model 1200 series HPLC machine with a fluorescence sensor [Model: RF20A] was utilized. The stationary phase was a 250-mm long Supelco® C18column, 4.6 mm, 5 m [Phenomenex, CA, USA] while the solvent system was a 60:20:20 combination of water, acetonitrile, and methanol. At ambient 25 °C temperature, extraction was done using an isocratic flow method with a flow rate of 1 ml min-1. The quantities of AFB1, AFB2, AFG1, and AFG2 measured by HPLC with fluorescence detection were assessed at five UV dosage levels [0, 1.22, 2.44, 3.66, and 4.88 J cm-2]. The UV radiation therapy was applied to a balanced design with three replicates for each treatment. Each specimen was distinct and was allocated to treatment at random [13], [28].

### 2.8 Rice quality assessment

Rice color is one of the important factors in determining the quality of rice. As a result, the impact of UV radiation on the color of rice was investigated [13], [28].

### 2.9 Evaluate the brightness of rice

The brightness of rice, which is one of its visual features, was used to determine whether or not the rice is impacted by varying levels of UV radiation. This is used to analyze rice samples that have been exposed to various UV treatments. The brightness of the sample was compared to the control group that had not been given any treatment. A DRK-100 light meter from the Derkson firm in China was used to evaluate the brightness. The relative brightness of the samples is assessed concerning these two standards after black and white standards are calibrated at zero and 100% brightness points [13], [28].

### 2.10 Evaluate rice aroma

Rice aroma was evaluated based on sensory and olfactory tests and compared with control samples and was rated as three strong, medium, and weak grades. After thoroughly mixing the samples, 1 g of the sample was weighed and placed in a test tube. Then 20 ml of distilled water was added to it and completely covered with aluminum foil in the tube to prevent steam and perfume from escaping. The test tube was placed in a boiling water bath for 10 min. After removing the tube from the boiling water bath and completely cooling the cooked rice, the aroma of the rice was evaluated by smell and compared to amberbu rice [with strong aroma] and Sepid Rud rice [without aroma] as a control. Because olfactory power is different in individuals, this test was performed by three trained individuals [28].

### 2.11 Amylose content determination

**Fig. 1:**
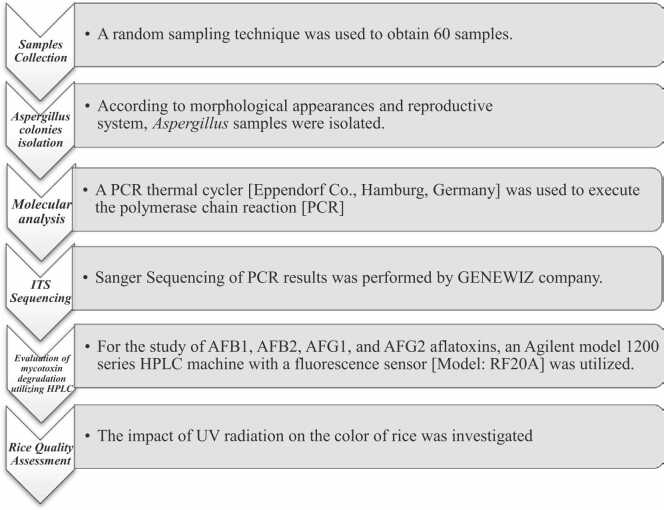
The graphical diagram represents the whole study.

Before and after receiving the radiation, the percentage of rice amylose was evaluated. As an external standard, a reference rice species with a certain quantity of amylose was utilized, and amylose levels were computed by comparing rice samples to a calibration chart. The absorbance was measured using an Agilent model 8453 diode array spectrophotometer with a wavelength range of 190–900 nm. Rice was crushed for 3 min in a high-speed IKA mill to quantify amylose, then 0.1 g of the sample was weighed and 1 ml of acetic acid and 9 ml of potassium hydroxide were added. It was placed in a boiling water bath for 15 min before being diluted with distilled water to a level of 100 ml after it had reached room temperature. 0.5 ml of this solution was mixed with 5 ml of distilled water, 0.1 ml of acetic acid, and 0.2 ml of 10% iodine solution; the quantity of rice amylose was then determined as a percentage utilizing calibration diagrams at four levels of 5%, 15%, 20%, and 30% amylose and absorption at 388 nm [13], [18], [28].Fig. 1.

### 2.12 Statistical analysis

The effects of various regimens on aflatoxins were assessed using one-way ANOVA with Tukey's HSD multiple comparison tests in the R statistical computer environment [R Development Core Team 2015]. Statistical results were reported as mean ± standard deviation. Statistically, a significant difference was determined at a threshold of 5% significance [*P* < *0.05*].

## 3 Results

### 3.1 Identification of aspergillus isolates from rice

**Fig. 2:**
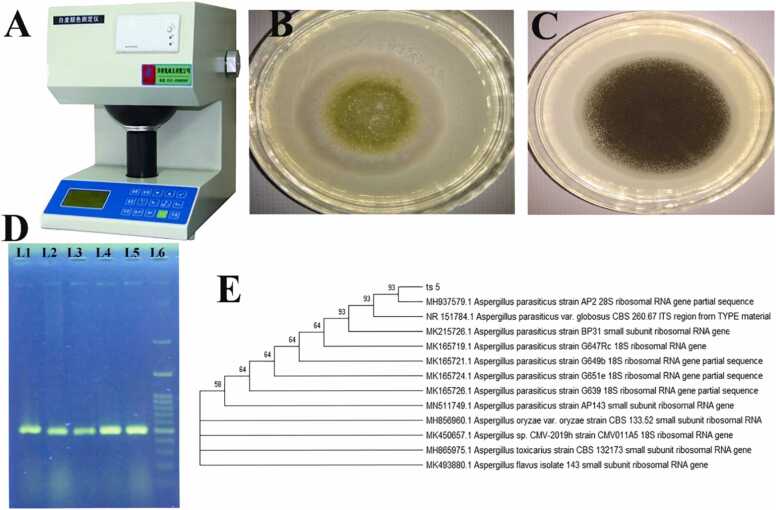
A: bench type collimated beam apparatus for medium-pressure UV system; B: Isolated image of *Aspergillus parasiticus*; C: *Aspergillus flavus* on the plate. D: The presence of ITS PCR products. Lane 1: Positive control, Lanes 2, 3, 4, 5: Experimental positive samples, Lane 6: 100 bp ladder.

Initial tests and assessment of the fungi's morphological parameters following initial purification and transfer to a new culture medium revealed that *Aspergillus* colonies were discovered in 33.3% [20 samples of 60] of rice samples [Fig. 2A, B, C]. Then, using molecular PCR analysis of the ITS gene, all 20/20 [100%] of the predicted isolates had a 500 bp band [Fig. 2D]. 12 strains [60%] were recognized as *Aspergillus flavus*, and 8 strains [40%] were detected as *Aspergillus parasiticus*, out of the 20 samples sequenced. The examined samples' ITS region sequences were 99–100% identical to the type strains of any gene in the NCBI database [https://blast.ncbi.nlm.nih.gov/Blast.cgi?PAGE TYPE=Blast Search]. The phylogenetic tree of the isolated strains also showed close affinity of the strains with Accession No MG430332.1 as *Aspergillus flavus* and Accession No MH937579.1 as *Aspergillus flavus* [Fig. 2D]. The phylogenetic tree showed the similarity of the isolated strains with the *Aspergillus parasiticus* and *Aspergillus flavus* fungal strains [Fig. 2E].

### 3.2 Results of mycotoxins assay

**Fig. 3:**
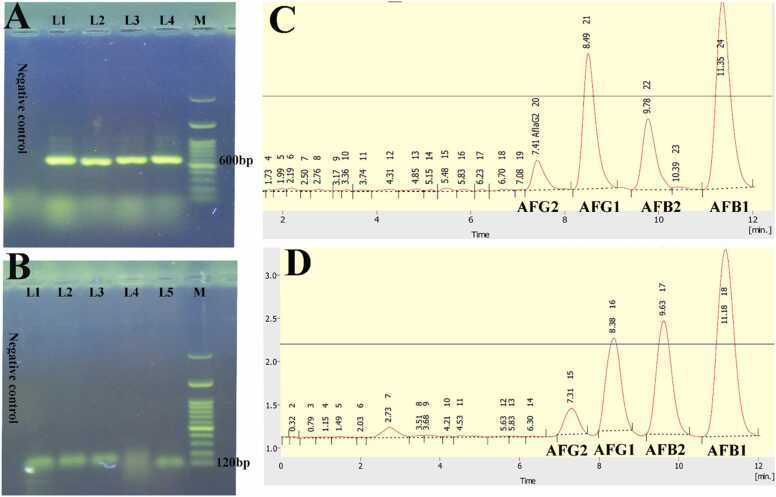
A: The presence of *ver-1*gene PCR products. Lane 1, 2: *Aspergillus parasiticus*, Lanes 3, 4: *Aspergillus flavus*; M: 100 bp ladder. B: The presence of *afl-R* gene PCR products. Lane 1, 2: *Aspergillus parasiticus*, Lanes 3, 4 and 5: *Aspergillus flavus*; M: 100 bp ladder. C: Chromatogram of standard working toxins used to prepare calibration diagrams Based on RT [Retention Time], the retention time was 7.41 for Afla G2, 8.49 for Afla G1, 9.78 for AflaB2, and 11.35 for AflaB1. D**:** Chromatogram of *Aspergillus flavus* strains capable of producing all four forms of aflatoxins [B1, B2, G1, G2]. Aflatoxin G2 took 7.31 min to withdraw, Aflatoxin G1 took 8.36 min, Aflatoxin B2 took 9.63 min, and aflatoxin B1 took 11.18 min. As can be observed in the image, aflatoxin B1 has a larger concentration than the others, indicating that this toxin is the first result of aflatoxins biosynthesis, with other forms of aflatoxins formed subsequently.
B) ***HPLC analysis of aflatoxin levels***

A) Results of molecular PCR assay for Ver-1 and afl-R Aflatoxin-specific biosynthetic genes
  A molecular PCR assay was used to look for the *Ver-1* and *afl-R* genes implicated in aflatoxin formation in Iranian rice. Aflatoxin-specific biosynthetic genes [*Ver-1*, *afl-R*] were positive in 12/12 [100%] *Aspergillus flavus* samples. Furthermore, In *Aspergillus flavus* strains, *afl-R* specific genes with 100% [8 samples] and *Ver-1* gene with 87.5% [7 samples] engaged in aflatoxin production [Figs. 3A, 2B].

Only one isolate of *Aspergillus* lacked toxin manufacturing capacity, according to HPLC measurements of toxin synthesis by isolated strains, which was compatible with PCR results. In AFB1, 87.5% [7 strains] of the *Aspergillus parasiticus* samples were over contaminated [5 ug/kg], and 1 sample did not produce in AFB1, AFB2, AFG1, and AFG2, respectively. AFB1 and AFB2 were also generated by 83.3% [10 strains] of *Aspergillus flavus* samples, with AFB1 levels being severe and AFB2 contamination being modest. The production capacity of AFG1 and AFG2 in *Aspergillus flavus* strains was 25%. All of these aflatoxins were less than 5 ug/kg, indicating mild contamination. According to the findings, three *Aspergillus parasiticus* strains [37.5%] were able to create all four types of aflatoxins, and aflatoxins B1, B2, G1, G2 were produced by 16.6% of *Aspergillus flavus* strains (Table 2).

**Table 2:** Results of aflatoxin measurements in specific fungal samples.

Strains	Sample ID	Aflatoxin B1-ug/kg	Aflatoxin B2-ug/kg	Aflatoxin G1-ug/kg	Aflatoxin G2-ug/kg
Aspergillus parasiticus	Test-002	23.5	6.3	1.5	1.3
	Test-007	18.9	5.4	2.4	1.8
	Test-008	20.9	6.6	1.1	1.7
	Test-012	ND	ND	ND	ND
	Test-016	25.8	5.7	ND	1.2
	Test-020	11.6	2.6	5.6	ND
	Test-021	22.4	8.1	ND	ND
	Test-023	19.7	4.7	ND	ND
Aspergillus flavus	Test-028	17.1	3.6	ND	ND
	Test-033	9.5	2.1	4.1	0.9
	Test-036	ND	ND	ND	ND
	Test-039	24.1	4.5	ND	ND
	Test-040	19.3	3.6	ND	ND
	Test-042	27.8	5.6	ND	ND
	Test-045	26.2	5.2	ND	ND
	Test-051	26.8	3.9	ND	ND
	Test-052	ND	ND	ND	ND
	Test-056	10.2	1.6	4.1	0.5
	Test-059	14.3	4.5	ND	ND
	Test-060	11.5	2.6	6.1	1.5

ND: Non-Detected.

Fig. 3C, D shows the chromatogram for the standard working toxins used to generate the calibration diagram, as well as the chromatogram for one of the isolated *Aspergillus flavus* strains that produce all four forms of aflatoxins B1, B2, G1, G2.

### 3.3 The effect of UV light on the decrease of Aflatoxin

**Fig. 4:**
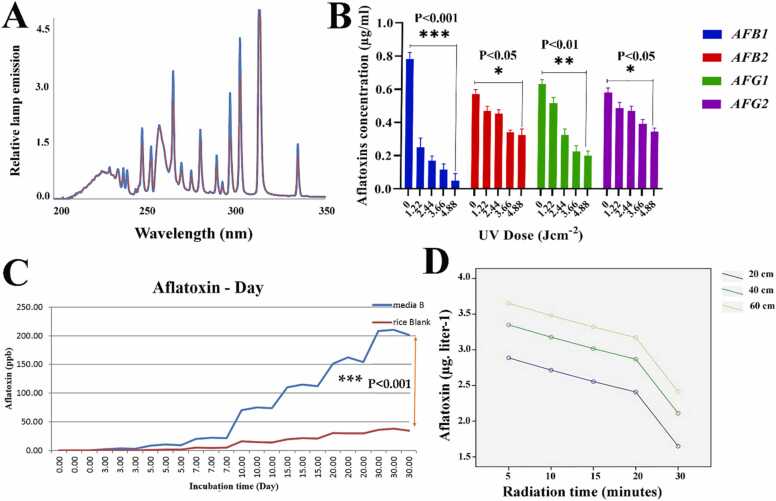
A: Medium-pressure lamp relative emission spectra. B: Degradation of aflatoxin. The results of three distinct studies are provided as mean percentage standard deviation. There is a significant difference in aflatoxin production at doses of 0, 1.22, 2.44, 3.66, and 4.88 [*P < *0.05*, **P < *0.01*, ***P < *0.001*]. C**:** The Relationship between aflatoxin production and incubation time. As can be observed, the rate of aflatoxin in pure culture media is significantly higher than in rice, and the quantity of aflatoxin increases with increasing growth time [* **P < 0.001].

The output of MP lights is seen in Fig. 4A. Aflatoxin reference values against the peak area standard curve were developed. The validated absorption intensity around 1027.536 ± 1.12 and 245.24 ± 3.15, 1021.136 ± 1.47 and 275.07 ± 3.44, 271.04 ± 0.23 and 78.60 ± 0.23, 251.37 ± 1.52 ng/ml for AFG1, AFG2, AFB2, and AFB1, respectively. R^2^ values for highest and lowest standard curves were 4.8 and 0.99 respectively, indicating that the observational results were well-fitted linearly. As shown in Fig. 4B, the amount of aflatoxin decreased with increasing UV radiation. AFG1 was lowered to 35.1, 48.2, 59.9, and 65.2%, significantly, at doses of 1.22, 2.44, 3.66, and 4.88 Jcm^−2^ [*P* < *0.01*]. Furthermore, at doses of 1.22, 2.44, 3.66, and 4.88 Jcm^−2^, AFB2 was shown to be reduced by 13.1%, 11.7%, 30.3%, and 28.9%. [*P* < *0.05*] (Fig. 4B). At a maximum dose of 4.88 Jcm^−2^, AFB1 was shown to be extremely susceptible to UV irradiation, with a > 70% decrease seen (*P* < *0.001*). As demonstrated in Fig. 4B, AFB1 and AFG1 were less resistant to UV irradiation than AFB2 and AFG2. AFB1 > AFG1 > AFB2 > AFG2 was the aflatoxins' susceptibility pattern. It's also important to note that the ambient temperature was raised during the trials in this investigation, and the aflatoxin compounds were clear. As a result, we may infer that heat-generating impacts were considerable, and that photochemical reaction played a significant role in the degradation and deactivation of aflatoxins. The impact of UV on the quantity of aflatoxin generated by *Aspergillus flavus* is shown in Fig. 4 C, which demonstrates that the quantity of aflatoxin generated was extremely tiny in the early days and increased with incubation time and fungal development. The quantity of mycotoxin in the identical samples dropped after being exposed to UV radiation of various intensities, as seen in the Fig. 4D. UV radiation can lower the level of toxins by more than 50%, according to the findings. In addition, the distance between the light source and the toxin was little in low doses of toxin, but it boosted the toxin's breakdown effectiveness greatly in large doses. In practically all treatments, the decrease levels followed the same pattern, however, the effect value [aflatoxin reduction] was larger in treatments that were closer [20 cm] to the samples. Aflatoxin decrease is shown in Fig. 4D as a function of UV irradiation time and distance from the light source. As the irradiation period increased, the level of aflatoxin B1 produced by *Aspergillus flavus* reduced, and this became more effective when the light source was situated 20 cm away [***P < *0.001*]. There were no significant differences in the distance in most treatments However, the rate of aflatoxin reduction was significant at a distance of 60 cm compared to 20 and 40 cm. Accordingly, a distance of 40 cm was effective to reduce the amount of aflatoxin [* *P < 0.01].

(a) D: Aflatoxin B1 level in *Aspergillus flavus* under the influence of UV irradiation time and distance to light source. (b) The amount of aflatoxin B1 of *Aspergillus flavus* decreases with increasing irradiation time, and this becomes more effective when it is placed at a distance of 20 cm from the light source [* **P < 0.001].

### 3.4 The qualitative effects of UV radiation on rice have not revealed any substantial alterations or flaws in rice quality

The results of sensory evaluations before cooking and after cooking rice did not show any significant changes and defects in the quality of rice after receiving ultraviolet light. At a distance of 20 cm from the light source, levels of aflatoxin were measured at 5, 10, 20, and 30-minute intervals. Humidity and amylose measures were taken before and after UV therapy, as shown in Table 3**.**

**Table 3:** The amylose content of rice before and after treatment with UV radiation.

	Blank		5 min		10 min		20 min		30 min	
DAY	HUMID	AMYLOSE	HUMID	AMYLOSE	HUMID	AMYLOSE	HUMID	AMYLOSE	HUMID	AMYLOSE
**0**	11.2	22	11.2	22	11.2	22	11.2	22	11.2	22
**3**	11.1	21.9	11	21.8	11	21.8	11.1	21.9	11.1	22
**5**	11.1	22	11	22	10.9	22	11	22	11.1	21.9
**7**	10.9	22	11	21.9	10.8	21.8	10.9	21.8	11	21.9
**10**	10.5	21.8	9.9	22	10.5	21.9	11	21.8	10.9	22
**15**	10	22.1	10	22	10	22	10.8	22	10.9	21.8
**20**	8	22	7.9	21.9	8	21.9	8.3	21.9	10.8	22
**30**	8.2	22	8	22	9.7	22	8.4	22	10.8	21.9

Along with taste, amylose is an important measure of rice quality. Slight differences were noticed in the collected data when compared to the control sample, but they were not significant and might have been related to sampling error. Even after the longest UV light treatment, rice quality was unaffected in terms of flavor, aroma, taste, humidity, and amylose. After undergoing long-term UV exposure after cooking, a sensory evaluation of rice was done, and no harmful effects on rice flavor or quality were identified (Table 4).

**Table 4:** Results of rice quality evaluation before and after receiving ultraviolet light treatment.

Treatment	Before treatment	After treatment
5 min at 20 cm light source	1. No abnormal taste and smell 2. Free of gelatinous and gypsum grains [maximum 5% by weight] 3. Has a natural smell and aroma of rice	1. No abnormal taste and smell 2. Free of gelatinous and gypsum grains [maximum 5% by weight] 3. Has a natural smell and aroma of rice
10 min at 20 cm light source	1. No abnormal taste and smell 2. Free of gelatinous and gypsum grains [maximum 5% by weight] 3. Has a natural smell and aroma of rice	a. No abnormal taste and smell 1. 2. Free of gelatinous and gypsum grains [maximum 5% by weight] 2. 3. Has a natural smell and aroma of rice
20 min at 20 cm light source	1. No abnormal taste and smell 2. Free of gelatinous and gypsum grains [maximum 5% by weight] 3. Has a natural smell and aroma of rice	1. No abnormal taste and smell 2. Free of gelatinous and gypsum grains [maximum 5% by weight] 3. Has a natural smell and aroma of rice
30 min at 20 cm light source	1. No abnormal taste and smell 2. Free of gelatinous and gypsum grains [maximum 5% by weight] 3. Has a natural smell and aroma of rice	1. No abnormal taste and smell 2. Free of gelatinous and gypsum grains [maximum 5% by weight] 3. Has a natural smell and aroma of rice

Only variations in rice relative humidity were noticed, which might be attributed to long-term storage in the incubator or UV light exposure. This decreasing tendency was seen in all samples with a single pattern, regardless of whether they were exposed to ultraviolet light for 5 min or got the most UV. It can be inferred that the decrease in humidity was not due to UV light, but as the incubation period increased from 5 to 15 and 20 days, the relative humidity decreased. Rice amylose did not change significantly when exposed to ultraviolet radiation [*P < 0.05*]. The brightness of rice did not alter much under the effect of UV light, according to the findings. This suggests that the rice's brightness did not alter much after being exposed to UV radiation. Table 5 summarized the brightness of rice in UV-treated samples and the control sample [which did not receive any light treatment]. The modifications were less than 1% in intensity.

**Table 5:** The amount of brightness in rice samples with different treatments of UV radiation compared to the control sample.

UV time [min]	Brightness repeat 1	Brightness repeat 2	Brightness repeat 3	Mean	STD deviation	% difference
10	80.1	80.02	80.05	80.05	0.343	0.838^**^
20	79.9	80.1	79.9	79.96	0.245	0.949^**^
30	79.8	79.9	79.85	79.85	0.451	1.0941^***^
Blank	80.6	80.7	80.9	80.73	0.368	ND

ND: Non-Detected. ** P < 0.01, ***P < 0.001.

## 4 Discussion

In this investigation, a toxin-producing fungus [*Aspergillus* genera], was collected from rice seeds. A large percentage of *Aspergillus flavus* [60%] was found, followed by *Aspergillus parasiticus* [40%] in the outcomes. *Aspergillus flavus* was shown to be the predominant contaminant in oilseeds, corn, wheat, cereals, and beans, according to various studies [25], [26], [27]. The findings of the Ranjbar et al. [2019] investigation was likewise in line with the findings of this study [28]. Rice and cereal products contain some relative pollution from the start due to saprophytes and ubiquitous toxicogenic fungi such as *Aspergillus*. The existence of spores and their multiplication during rice shipping, as well as contamination of the storage system with these fungi, can cause contamination to spread. Drought stress, as discovered by Magnussen [2013] and Ranjbar [2019], is one of the variables boosting seed sensitivity to *Aspergillus* and aflatoxin contamination [26], [28]. The findings of this research also imply that the incidence of these organisms might be enhanced with high-temperature situations and prolonged storage duration. According to research published in 2019, the prevalence of aflatoxins in food is affected by a variety of factors including the season, temperature, humidity, target location, collecting technique, storing, and preparation [29]. Another study looked at the effects of climate and incubation temperature on *Aspergillus flavus* development and AFB1 generation on seeds, finding that the flavus strain can grow over a wide range of temperatures [15–37 °C], while aflatoxin production occurs at a higher temperature range [25–37 °C] [30]. The occurrence of *Aspergillus* did not imply the existence of mycotoxin in this investigation, and the occurrence of mycotoxin did not indicate the existence of *Aspergillus*.

In this investigation MPUV lamps were employed, which have a radiation pattern covering 200–360 nm UV, allowing for multi-exposure to several spectra. The LC test resulted in a reduction in the peak, which was utilized to assess aflatoxins removal. At dosages of 1.22, 2.44, 3.66, and 4.88 Jcm^−2^, AFG1 was considerably reduced to 35.1%, 48.2%, 59.9%, and 65.2% [*P < 0.01*]. Additionally, AFB2 was found to be decreased by 13.1%, 11.7%, 30.3%, and 28.9% at dosages of 1.22, 2.44, 3.66, and 4.88 Jcm^−2^. [*P < 0.05*]. AFB1 was found to be particularly vulnerable to UV irradiation at a maximal level of 4.88 Jcm^−2^, with a > 70% drop [*P < 0.001*]. AFB1 significantly absorbs UV light spectrum of 222, 265, and 362 nm [31], many of which are produced by intermediate lights, resulting in greater degrading efficiency when opposed to older single wavelength UV light emission. Radiation exposure therapies have been shown to heat up materials given the intensity of the brightness. Radiation exposure was used to separate the role of temperature changes on aflatoxin decomposition by keeping liquid temps under 5– 8 °C during procedures. Mycotoxins have great thermodynamic tolerance, and only at degrees over 160 °C do they begin to degrade [14], [32]. To minimize mycotoxins, Udomkun et al. [33] adopted a pulsed light (PL) method. The visible and infrared sections of PL, according to the researchers, won't be contributing to the photochemical reactions that destroy aflatoxins. The destruction of mycotoxin was primarily influenced by the UV spectral range and PL light intensity. The intermediate UV lights are expected to behave similarly. According to the findings, AFB1 and AFG1 were sensitive to UV irradiation, although AFB2 and AFG2 were more robust. AFB1 > AFG1 > AFB2 > AFG2 was the order of aflatoxins' susceptibility. Such variations in decomposition profile are consistent with previous research [34] and can be related to the chemical compositions of AFB1 and AFG1, particularly a double link between both the Eight and Nine carbon atoms of the furan ring in AFB1 and AFG1, in contrast to AFB2 [35]. Maybe the C8–C9 dual-link in the port furan ring of AFB1 renders it much more susceptible to photo-degradation/photo-oxidation than AFB2, which lacks such a double bridge and is, therefore, more robust [36]. It is indeed important to note that the ambient temp remained constant throughout the trials, and the aflatoxin fluids were visible. As a result, we may conclude that heat-generating impacts were minor, and that photochemical reactions were more important in the degradation and deactivation of aflatoxins. To our knowledge, this is one of the earliest demonstrations of a light-based technique capable of destroying high-temperature mycotoxins [especially AFB1] and, more importantly, nearly completely inactivating their effects. The absorption spectrum was used to identify deteriorated compounds, which were then validated utilizing LC-MS/MS particular chemical analyses. Selected ion monitoring (SIM) chromatograms were used to assess the peak regions of mycotoxin and degradation products for the standards and treated samples. Anjum et al. discovered AFB1 UV-degraded compounds *m/z* 331 and *m/z* 317 [37], [38], [39], [40], [41], while Iram et al. discovered AFB2-degraded compound *m/z* 301 [38], [39], [40]. In the present study, the effect of UV therapy on rice quality was also investigated. According to the results, there was no significant change in brightness (Table 5), amylose, and taste of rice (Table 4).

## 5 Conclusion

UV radiation was examined as a low-risk and effective approach to reduce food contamination [rice] in this study, and the findings revealed that this technology may be utilized to improve quality and control *Aspergillus spp*. contamination in products such as rice. This decontamination procedure can lower the quantity of fungus and bacteria generally, in addition to decreasing the number of probable aflatoxins in rice. This sort of technology is crucial since it may be used at any point throughout the manufacturing, processing, and distribution processes. According to this study, 30 min and 20 cm distance of light source has the biggest impact on isolated species, and while 5, 10 and 20-minute times have been able to reduce the amount of *Aspergillus flavus* and *Aspergillus parasiticus* below the permissible limits in some cases. UV light with lower energy and lower danger can help minimize aflatoxin contamination in food.

## Disclaimer

The findings and conclusions in this report belong to the corresponding author.

## Funding

Not applicable.

## CRediT authorship contribution statement

Farideh Tabatabaee Yazdi designed and carried out the experiments and drafted the manuscript, Hamed Faraji, collected the review literature, drafted and analyzed the data, Nematollah Razmi analyzed and drafted the manuscript, Farideh Tabatabaee Yazdi and Hamed Faraji thoroughly edited the manuscript.

## Declaration of Competing Interest

The authors declare that they have no known competing financial interests or personal relationships that could have appeared to influence the work reported in this paper.
